# The Interplay Between TGF-β Signaling and Cell Metabolism

**DOI:** 10.3389/fcell.2022.846723

**Published:** 2022-03-09

**Authors:** Huidong Liu, Ye-Guang Chen

**Affiliations:** The State Key Laboratory of Membrane Biology, Tsinghua-Peking Center for Life Sciences, School of Life Sciences, Tsinghua University, Beijing, 100084, China

**Keywords:** TGF-β signaling, Smad, glucose metabolism, lipid metabolism, amino acid metabolism

## Abstract

The transforming growth factor-β (TGF-β) signaling plays a critical role in the development and tissue homeostasis in metazoans, and deregulation of TGF-β signaling leads to many pathological conditions. Mounting evidence suggests that TGF-β signaling can actively alter metabolism in diverse cell types. Furthermore, metabolic pathways, beyond simply regarded as biochemical reactions, are closely intertwined with signal transduction. Here, we discuss the role of TGF-β in glucose, lipid, amino acid, redox and polyamine metabolism with an emphasis on how TGF-β can act as a metabolic modulator and how metabolic changes can influence TGF-β signaling. We also describe how interplay between TGF-β signaling and cell metabolism regulates cellular homeostasis as well as the progression of multiple diseases, including cancer.

## Introduction

Comprising 33 members in mammalian cells, the transforming growth factor-β (TGF-β) superfamily is distinct from other cytokines owning to its more widespread and pleiotropic effects (Morikawa et al., 2016). The TGF-β signaling pathway contributes to a broad range of physiological and pathological processes, and its key roles in development, immunity, wound healing, cancer, fibrosis, skeletal and cardiac diseases have been extensively studied (Massague, 2008; Wu and Hill, 2009; Dobaczewski et al., 2011; Travis and Sheppard, 2014; Meng et al., 2016; Morikawa et al., 2016; Salazar et al., 2016; Kiritsi and Nystrom, 2018; Derynck et al., 2020). A plethora of cellular activities, including cell proliferation, differentiation, apoptosis, adhesion and migration, are controlled by TGF-β superfamily members in a context-dependent manner (Feng and Derynck, 2005; Moustakas and Heldin, 2009; Massague, 2012; David and Massague, 2018). Although cellular responses to TGF-β signaling are mainly induced via its transcriptional regulation of genes (Massague and Chen, 2000; Massague and Wotton, 2000), other means have been recognized for TGF-β signaling to shape cell behavior, such as epigenetic modification, mRNA splicing and miRNA expression (Derynck and Budi, 2019). In addition, accumulating evidence indicates that TGF-β signal can also remodel cell metabolism.

As a network of chemical reactions essential for sustaining life, metabolism has long been centered in energy provision, building of blocks for biomacromolecules and elimination of compounds that are otherwise toxic to the organism. Studies in the past decades, especially with the aid of metabolomics, have further unraveled the profound interactions between metabolism and the regulation of protein activity and genes expression (Rinschen et al., 2019). Metabolic substrates, beyond serving as “ingredients” or biomarkers, are able to modify the chromatin structure and regulate gene expression (Li et al., 2018). On the other hand, metabolic enzymes, in response to signaling cues, can fulfill many moonlighting functions other than catalyzing (Xu et al., 2021). Therefore, these non-metabolic roles of metabolites and metabolic enzymes have been shown to play a critical role in signal transduction.

In this review, we discuss the current knowledge of how TGF-β signaling functions by altering various facets of cell metabolism and how metabolic changes can result in modulation of TGF-β signaling, thereby affecting an array of cellular processes. Such interplay between TGF-β signaling and cell metabolism is thought to be instrumental in maintaining homeostasis, and its aberration contributes to disease development. Due to the large number of TGF-β superfamily members, the scope of this review is restricted to the TGF-β ligands (TGF-β1, 2 and 3), which have been most extensively studied.

## Basics of the TGF-β Signaling Pathway

Based on the similarities in protein sequence and structure, the mammalian TGF-β members, with a few exceptions, can be classified into three major groups: the TGF-β family, the inhibin/activin family and the BMP (bone morphogenic protein)/GDF (growth and differentiation factor) family (Morikawa et al., 2016). The TGF-β family consists of TGF-β1, 2 and 3 that have largely redundant functions. Each isoform contains nine highly conserved cysteine residues, mediating the formation of inter- or intramolecular disulfide bonds that interlock two TGF-β polypeptides as a dimer (Hinck et al., 2016). The dimeric TGF-β ligand associates with the pro-region-derived latency-associated peptide (LAP) and a latent TGF-β binding protein (LTBP) and forms a large latent complex (LLC), which is trapped in the extracellular matrix (ECM) (Robertson and Rifkin, 2016). Activation of TGF-β ligands is mediated by different proteins in various tissues, serving as a way to ensure the precision of signal presentation (Rifkin, 2005).

**FIGURE 1:**
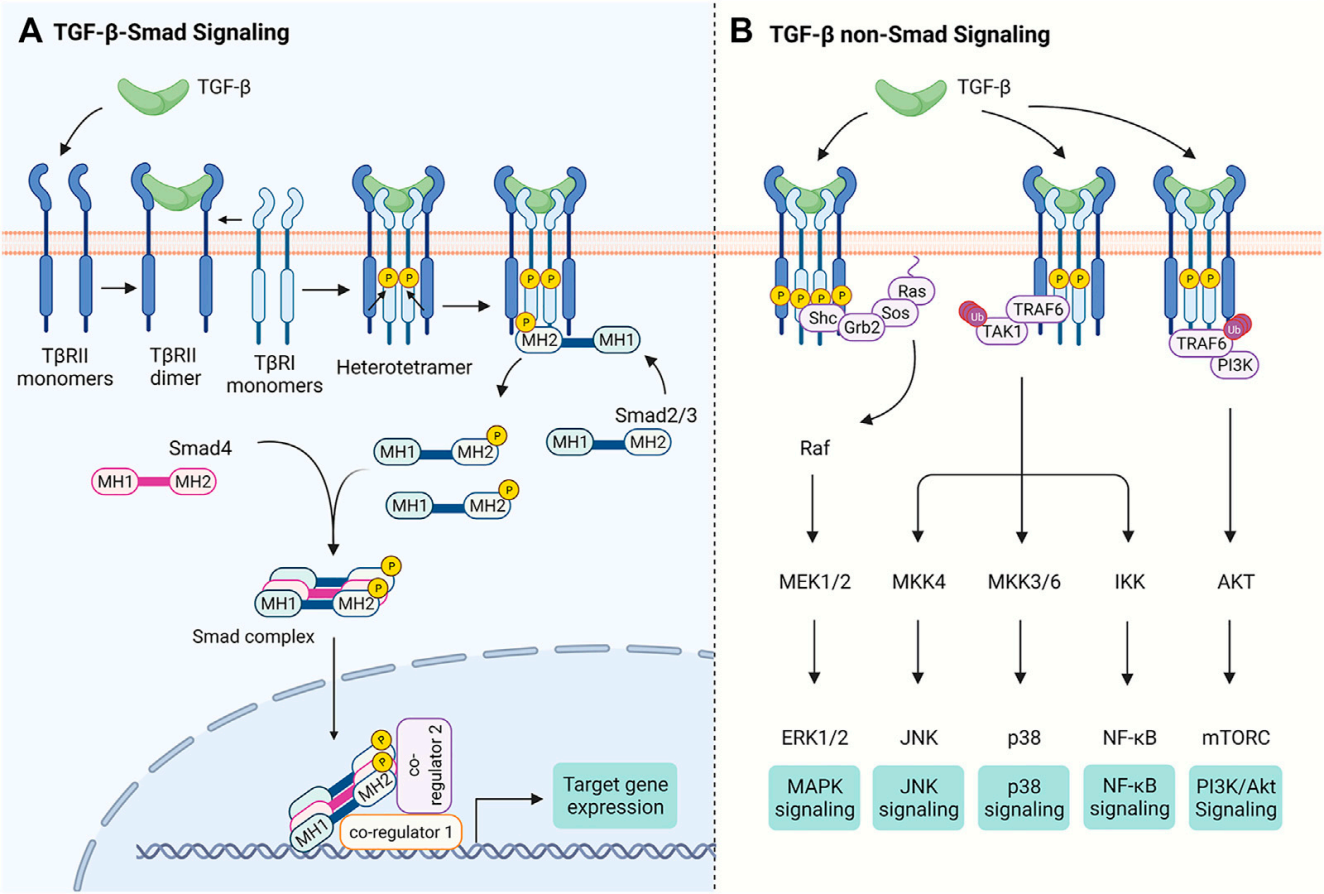
The TGF-β Signaling Pathways. Binding of TGF-β to TβRII leads to the tetramer assembly of monomeric TβRII and TβRI receptors. **(A)** In Smad-dependent TGF-β signal transduction, TβRII trans-phosphorylates TβRI and activates its kinase activity, which in turn phosphorylates Smad2/3 at the C-terminal tail. Phosphorylated Smad2/3 form a trimeric complex with Smad4 and is translocated into the nucleus. The Smad complex binds DNA via their MH1 domains and controls gene expression through interacting with other transcription co-regulators. **(B)** TGF-β receptors trigger non-Smad signaling pathways. For instance, TGF-β receptors have been reported to recruit Shc, Grb2 and Sos to activate Ras, thereby initiating MAPK signaling. TGF-β receptors also activate TAK1 through TRAF6, which is required for TGF-β-induced JNK, p38 and NF-κB activation. It has been proposed that interaction of TRAF6 with TβRI also leads to PI3K/Akt activation. Figure is created with Biorender.com.

Once activated, the dimeric TGF-β initiates signaling by promoting the assembly of two type I (TβRI) and two type II (TβRI) transmembrane receptors (Hata and Chen, 2016) (Figure 1). In the absence of TGF-β ligands, both TβRI and TβRII exist as monomers (Zhang et al., 2009; Zhang et al., 2010), although early studies reported that they exist as homodimers (Chen and Derynck, 1994; Henis et al., 1994; Gilboa et al., 1998), most likely due to their overexpression. Both of TβRI and TβRII possess Ser/Thr kinase activity in the cytoplasmic domain. Ligand binding results in the tetramer receptor complex formation with two TβRI and two TβRII, in which TβRI is activated via phosphorylation of Thr and Ser residues in its GS domain (TTSGSGSG) by the constitutively active TβRII (Wrana et al., 1994). The phosphorylation-induced conformational change activates the TβRI kinase that relays the signal to the effector Smad proteins (Huse et al., 1999; Huse et al., 2001; Chaikuad and Bullock, 2016; Hata and Chen, 2016) (Figure 1A).

There are three subgroups of Smad proteins: the receptor-activated Smads (R-Smads, Smad2/3 for TGF-β/activin/inhibin receptors and Smad1/5/8 for BMP/GDF receptors), the common-mediator Smad (Co-Smad, i.e., Smad4) that interacts with R-Smads, and the inhibitory Smads (I-Smads, Smad6 and Smad7). Both R- and Co-Smads propagate signals, while I-Smads dampen the signal transduction (Hata and Chen, 2016). All R-Smad proteins contain a highly conserved C-terminal MH2 domain that, via an inner L3 loop, engages in Smad-receptor and Smad-Smad interactions (Lo et al., 1998). The conserved N-terminal MH1 domain in R-Smads and Co-Smad has a nuclear localization signal and a DNA-binding β-hairpin (Hata and Chen, 2016; Chaikuad and Bullock, 2016; Shi et al., 1998; Hill, 2016). Upon activation of TβRI kinase activity, Smad2/3 is phosphorylated at two serine residues in the SSXS motif and subsequently is dissociated from the TβRI kinase domain, forming a trimeric Smad complex composed of two Smad2/3 and one Smad4 (Hata and Chen, 2016; Chaikuad and Bullock, 2016; Kawabata et al., 1998; Chacko et al., 2004; Xu et al., 2016). This Smad complex is then accumulated in the nucleus and acts as a transcription factor to regulate contextual expression of target genes through collaboration with diverse co-factors (Moustakas and Heldin, 2009; Massague et al., 2005) (Figure 1A). TGF-β ligands can also signal independently of Smad proteins through crosstalk with other signaling pathways (see Zhang, 2017; Derynck and Budi, 2019) (Figure 1B).

While it is clear that TGF-β signaling targets genes related to cell cycle progression, ECM production and epithelial-mesenchymal transition (EMT), a panoramic view of metabolic genes whose transcription directly controlled by TGF-β signaling are not attained. It remains even more obscure precisely how metabolic changes regulate the TGF-β signal transduction. In the following sections, we will illustrate the interplay between TGF-β signaling and multiple aspects of cell metabolism with a discussion on their important physiological or pathological roles in mammalian cells.

## TGF-β Signaling and Glucose Metabolism

**FIGURE 2:**
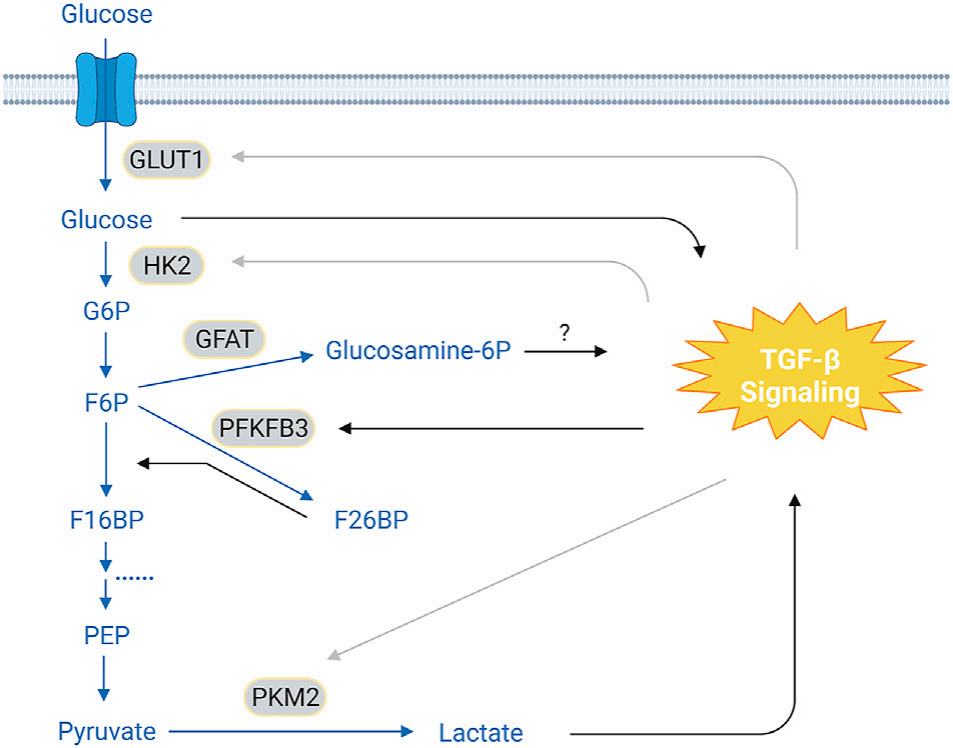
Crosstalk Between TGF-β Signaling and Glycolysis. In the glycolytic pathway, glucose is converted to pyruvates via a cascade of enzymatic reactions. It has been reported that TGF-β signaling can either increase or decrease the expression of GLUT1 and HK2, depending on the cell types. This cell type-context effect is also seen on PKM2, an enzyme that catalyzes pyruvate into lactate. TGF-β signaling upregulates PFKFB3, leading to increased F26BP levels, which, in turn, accelerate conversion of F6P to F16BP. It has been suggested that glucose and lactate can promote TGF-β signaling; and inhibition of GFAT prevents glucose-induced expression of TGF-β ligands, implying a potential role of glucosamine-6-phosphotase in mediating this process. The conversion of F16BP to pyruvate has been omitted for clarity. Blue texts and arrows, glycolysis and its branches; gray arrows, cell-type dependent effect. Abbreviations: G6P, glucose-6-phosphate; F6P, fructose-6-phosphate; F16BP, fructose-1,6-biphosphate; F26BP, fructose-2,6-biphosphate; PEP, phosphoenolpyruvate; GLUT1, glucose transport 1; HK2, hexokinase 2; GFAT, glutamine:fructose-6-phosphate aminotransferase; PFKFB3, 6-phosphofructo-2-kinase/fructose-2,6-biphosphatase 3; PKM2, pyruvate kinase M2. Figure is created with BioRender.com.

The first evidence that TGF-β regulates glucose metabolism perhaps comes from work on Swiss mouse 3T3 cells demonstrating TGF-β treatment upregulates *Glut1* (glucose transporter type 1) mRNA level and increases glucose uptake (Kitagawa et al., 1991). This observation is later reproduced in rat glomerular mesangial cells and is associated with excessive glucose uptake-induced overproduction of ECM proteins (Inoki et al., 1999), which is a hallmark of diabetic nephropathy. In a different model using mouse normal mammary gland (NMuMG) cells to study TGF-β-induced EMT, however, Glut1 expression is reduced at both the protein and mRNA levels during short-term TGF-β exposure but is later restored, which may be explained by differential effects of TGF-β on proliferation of epithelial and mesenchymal cells through regulation of glucose uptake (Nilchian et al., 2020). In mesangial cells, high glucose can potently increase autocrine secretion of TGF-β (Ziyadeh et al., 1994; Kolm et al., 1996; Kolm-Litty et al., 1998) (Figure 2). It seems that a positive feedback loop, in which elevated glucose levels stimulate TGF-β production and TGF-β, in turn, enhances glucose uptake, may pathologically contribute to the progression of diabetic nephropathy. Interestingly, production of TGF-β induced by high glucose is impaired by inhibition of Gfat (Glutamine:fructose-6-phosphate aminotransferase, the rate-limiting enzyme that converts fructose-6-phosphate into glucosamine-6-phosphate) (Kolm-Litty et al., 1998), suggesting a potential role of glucosamine-6-phosphotse in regulating TGF-β expression (Figure 2). In addition to promote TGF-β ligand production, high glucose is shown to increase cell membrane levels of both TβRI and TβRII and to induce latent-TGF-β activation by matrix metalloproteinases, leading to activation of the Akt-mTOR pathway and consequently causing cell hypertrophy in fibroblasts and epithelial cells (Wu and Derynck, 2009).

TGF-β signaling also regulates other components in the glycolytic pathway (Figure 2; Table 1A). For example, TGF-β treatment significantly decreases hexokinase 2 (HK2) expression in murine thymic-derived Tregs (Priyadharshini et al., 2018; Chen et al., 2020). However, HK2 levels are slightly increased in TGF-β-treated articular chondrocytes from patients with osteoarthritis (Wang et al., 2018). TGF-β stimulation also specifically increases HK2 abundance in murine and human lung fibroblasts, which is required for profibrotic actions of TGF-β possibly through upregulating YAP/TAZ protein levels by an unknown mechanism (Yin et al., 2019). These results together suggest a cell-type dependent effect of TGF-β signaling on HK2 regulation. Phosphofructokinase 2 (PFK2), an enzyme that generates fructose-2,6-biphosphate that allosterically activates phosphofructokinases, is overexpressed in many cancer cells. TGF-β induces PFK2 expression in glioblastoma and pancreatic cancer cells (Rodriguez-Garcia et al., 2017; Yalcin et al., 2017), which is required for activation of SNAI1 transcription and promotes cell invasion (Yalcin et al., 2017). In SW480 colon cancer cells, increased pyruvate kinase M2 (PKM2) expression by TGF-β and EGF has been reported to be indispensable for EMT (Hamabe et al., 2014). In podocytes, the interaction of Smad4 with PKM2 interrupts the active PKM2 tetramer and reduces glycolysis activity (Li et al., 2020).

**TABLE 1:** TGF-β-induced metabolic changes.

Target	Effect on metabolism	Implication	Cell type	Reference
A. Glucose metabolism				
GLUT1	Glucose uptake ↑	Proliferation	Fibroblasts	Kitagawa et al. (1991)
	Glucose uptake ↑	ECM production	Mesangial cells	Inoki et al. (1999)
	Glucose uptake ↓	Antiproliferation, EMT	Mammary epithelial cells	Nilchian et al. (2020)
HK2	Glycolysis ↓	NA	Thymic Treg cells	Priyadharshini et al. (2018); Chen et al. (2020)
	Glycolysis ↑	Osteoarthritis	Articular chondrocytes	Wang et al. (2018)
	Glycolysis ↑	Fibrosis	Lung fibroblasts	Yin et al. (2019)
PFKFB3	Glycolysis ↑	Invasion	Glioblastoma, pancreatic cancer cells	Rodriguez-Garcia et al. (2017); Yalcin et al. (2017)
PKM2	Aerobic glycolysis ↑	EMT	Colon cancer cells	Hamabe et al. (2014)
	Glycolysis ↓	Diabetic injury	Podocytes	Li et al. (2020)
PDC	OXPHOS ↓	Kidney injury	Fibroblasts	Smith and Hewitson, (2020)
Fumarase	Moonlighting effect	Cell cycle arrest	Lung cancer cells	Chen et al. (2019)
COX IV	OXPHOS ↓	Cell cycle arrest	Lung epithelial cells	Yoon et al. (2005)
COX I	OXPHOS & fatty acid oxidation ↑	EMT	Breast cancer cells	Liu et al. (2020)
ATP synthase	OXPHOS ↓	Impaired tumor immunity	Effector memory T cells	Dimeloe et al. (2019)
G6PC3	Gluconeogenesis ↑	HSC differentiation	Zebrafish embryonic cells	Zhang et al. (2018)
B. Lipid metabolism				
SCD	Unsaturated fatty acid synthesis ↑	NA	Epithelial cells and fibroblasts	Samuel et al. (2002)
FASN	Fatty acid synthesis ↓	EMT	Breast and lung cancer cells	Jiang et al. (2015); Yang et al. (2016); Liu et al. (2020)
SPHK1	Sphingosine-1P ↑	NA	Fibroblasts	Yamanaka et al. (2004)
	Sphingosine ↑	Dysfunctional placentae	Choriocarcinoma cells	Chauvin et al. (2015)
ASAH1	Sphingosine ↑	Dysfunctional placentae	Choriocarcinoma cells	Chauvin et al. (2015)
SHIP	PI(3, 4, 5)P_3_ ↓	Apoptosis	Immune cells	Valderrama-Carvajal et al. (2002)
CYP24A1	1,25(OH)_2_D_3_ ↓	Impaired host defense	Airway epithelial cells	Schrumpf et al. (2020)
Ceramide	Ceramide ↑	ECM production	Fibroblasts	Sato et al. (2003)
	Ceramide ↓	Cell survival	Fibroblasts	Chen et al. (2003)
Lipid droplet	Fatty acids storage ↑	EMT, invasion	Acidosis-adapted cancer cells	Corbet et al. (2020)
	Fatty acids storage ↑	Impaired tumor immunity	Dendritic cells	Trempolec et al. (2020)
	Fatty acids storage ↑	Impaired tumor immunity	Macrophages	Bose et al. (2019)
C. Amino acid metabolism				
P4HA3	Reprogrammed amino acid metabolism	EMT	Lung cancer cells	Nakasuka et al. (2021)
GLS1	Glutamine anaplerosis ↑	NA	Hepatocellular carcinoma cells	Soukupova et al. (2017)
	Glutaminolysis ↑	EMT	Breast cancer cells	Lee et al. (2016)
	Glutaminolysis ↑	Fibrosis	Myofibroblasts	Bernard et al. (2018)
	Glutaminolysis ↑	Fibrosis	Fibroblasts	Choudhury et al. (2020)
ARG1	Polyamine synthesis ↑	Immunosuppression	Dendritic cells	Mondanelli et al. (2017)
	Polyamine synthesis ↑	Impaired tumor immunity	Macrophages	Boutard et al. (1995)
	Polyamine and proline synthesis ↑	ECM production	Vascular smooth muscle cells	Durante et al. (2001)
IDO1	Moonlighting effect	Immunosuppression	Dendritic cells	Mondanelli et al. (2017)
	Moonlighting effect	Self-tolerance	Dendritic cells	Pallotta et al. (2011)
	Tryptophan metabolism ↑	NA	Fibroblasts	Yuan et al. (1998)
ATF4	Serine-glycine synthetic pathway ↑	ECM production	Lung fibroblasts	Selvarajah et al. (2019)
SLC3A2	Leucine uptake ↓	Cell cycle arrest	Mammary epithelial cells	Loayza-Puch et al. (2017)
P5CS, PYCR1/2	Proline synthesis ↑	Fibrosis	Fibroblasts	Schworer et al. (2020)
D. Redox, polyamine and other aspects of cell metabolism				
NOX4	ROS ↑	Fibrosis, cancer	Multiple tissues of origin	Cucoranu et al. (2005); Sturrock et al. (2006); Carmona-Cuenca et al. (2008); Michaeloudes et al. (2011); Boudreau et al. (2012)
Glutathione	Glutathione metabolism ↓	Fibrosis	Multiple tissues of origin	see Liu and Gaston Pravia, (2010) for review
	Glutathione metabolism ↑	Drug resistance	Squamous cell carcinoma cells	Oshimori et al. (2015)
ODC1	Polyamine synthesis ↓	NA	Leukemia cells	Motyl et al. (1993)
	Polyamine synthesis ↑	NA	Myofibroblasts	Blachowski et al. (1994)
AMD1	Polyamine synthesis ↓	NA	Leukemia cells	Motyl et al. (1993)
	Polyamine synthesis ↑	NA	Myofibroblasts	Blachowski et al. (1994)
Putrescine	Putrescine ↑	Impaired tumor immunity	Macrophages	Boutard et al. (1995)
	Putrescine ↑	ECM production	Vascular smooth muscle cells	Durante et al. (2001)
Spermidine	Spermidine ↑	Self-tolerance	Dendritic cells	Mondanelli et al. (2017)
PNPO	Vitamin B6 metabolism ↑	Cell proliferation	Ovarian cancer cells	Zhang et al. (2017b)

GLUT1, Glucose Transporter 1; HK2, Hexokinase 2; PFKFB3, Fructose-2,6-Biphosphatase 3; PKM2, Pyruvate Kinase M2; PDC, Pyruvate Dehydrogenase Complex; OXPHOS, oxidative phosphorylation; COX, Cytochrome c Oxidase; G6PC3, Glucose-6-Phosphatase Catalytic Subunit 3; SCD, Stearoyl-CoA Desaturase; FASN, Fatty Acid Synthase; SPHK1, Sphingosine Kinase 1; ASAH1, N-Acylsphingosine Amidohydrolase 1; SHIP, SH2 domain-containing 5′ Inositol Phosphatase; CYP24A1, Cytochrome P450 Family 24 Subfamily A Member 1; P4HA3, Prolyl 4-Hydroxylase Subunit Alpha 3; GLS, Glutaminase; ARG1, Arginase 1; IDO1, Indoleamine 2,3-Dioxygenase 1; ATF4, Activating Transcription Factor 4; SLC3A2, Solute Carrier Family 3 Member 2; P5CS, Delta-1-Pyrroline-5-Carboxylate Synthase; PYCR1/2, Pyrroline-5-Carboxylate Reductase 1/2; NOX4, NADPH Oxidase 4; ROS, reactive oxygen species; ODC1, Ornithine Decarboxylase 1; AMD1, Adenosylmethionine Decarboxylase 1; PNPO, Pyridoxamine 5′-Phosphate Oxidase; ↑, increase. ↓, decrease.

Lactate, the product of anaerobic glycolysis generated from pyruvate, appears to positively modulate TGF-β signaling (Figure 2; Table 2A). For instance, lactate induces TGF-β2 expression in glioma cells and knockdown of lactate dehydrogenase A (LDHA), an enzyme that catalyzes lactate production, downregulates TGF-β2 levels (Baumann et al., 2009). Lactate generated during exercising is associated with increased bioactive TGF-β concentration in rat cerebrospinal fluid (Yamada et al., 2012). Consistently, injection of lactate into mice results in elevated serum TGF-β2 levels, and incubation of adipocytes with lactate causes increased TGF-β2 concentrations in the media (Takahashi et al., 2019), though the underlying mechanism remains to be determined.

**TABLE 2:** Modulation of TGF-β signaling by metabolic changes.

Metabolic Event	Effect on TGF-β signaling	Outcome	Reference
A. Glucose metabolism			
High glucose	TGF-β production/ secretion ↑	ECM production	Ziyadeh et al. (1994); Kolm et al. (1996); Kolm-Litty et al. (1998)
		Cell hypertrophy	Wu and Derynck, (2009)
	TβRI/II membrane levels and TGF-β bioactivity ↑	Cell hypertrophy	Wu and Derynck, (2009)
Inhibition of GFAT	TGF-β production/ secretion ↓	ECM reduction	Kolm-Litty et al. (1998)
Increased lactate	TGF-β production/ secretion ↑	Cell migration	Baumann et al. (2009)
		Energy expenditure	Yamada et al. (2012); Takahashi et al. (2019)
B. Lipid metabolism			
Increased β-hydroxybutyrate	*TGFB* expression ↑	ECM production	Guh et al. (2003)
Overexpression of SGMS1	*TGFBRI* expression ↓	EMT inhibition	Liu et al. (2019)
Treatment of ceramide	TβRI/II membrane levels ↓	Inhibition of cell migration/invasion	Gencer et al. (2017)
Treatment of S1P	p-Smad2 levels ↑	NA	Yamanaka et al. (2004)
Loss of Nsdhl	*Tgfb1* expression and TGF-β production/ secretion ↑	EMT	Gabitova-Cornell et al. (2020)
Expression of NSDHL	TβRII levels ↑	Metastasis	Chen et al. (2021)
Treatment of RA with TGF-β	Smad3 and p-Smad3 levels ↑	Treg differentiation	Xiao et al. (2008)
Treatment of vitamin D	p-Smad2 levels ↓	Fibrosis inhibition	Halder et al. (2011); Beilfuss et al. (2015)
Activation of VDR	Smad3 binding to target DNA ↓	Fibrosis inhibition	Ding et al. (2013)
C. Redox, polyamine and other aspects of cell metabolism			
Depletion of intracellular PA	TβRI/II levels ↑ Total nuclear Smad3, 4 levels ↑	Cell cycle arrest	Patel et al. (1998); Rao et al. (2000)
			Liu et al. (2003)
Secretion of adenosine	p-Smad2/3 levels ↓	ECM reduction	Vasiukov et al. (2020)
Downregulation of XDH	TGF-β production/ secretion and p-Smad2/3 levels ↑	EMT	Chen et al. (2017)

GFAT, Glutamine:Fructose-6-Phosphate Aminotransferase; SGMS1, Sphingomyelin Synthase 1; S1P, Sphingosine-1-Phosphatase; NSDHL, NAD(P) Dependent Steroid Dehydrogenase-Like; RA, Retinoic Acid; VDR, Vitamin D Receptor; PA, Polyamine; XDH, xanthine dehydrogenase. ↑, increase; ↓, decrease.

When oxygen is plentiful, pyruvate generally enters the TCA cycle, and most ATP is produced via oxidative phosphorylation (OXPHOS). TGF-β signaling has been shown to attenuate pyruvate dehydrogenase complex (PDC) activity in fibroblasts from injured kidneys and reduces free acetyl-CoA levels (Smith and Hewitson, 2020). TGF-β also causes phosphorylation of fumarase at T90 via the p38 pathway (Chen et al., 2019). Although the phosphorylated fumarase seems to retain normal catalytic activity, it gains non-metabolic functions and can shuttle into the nucleus to activate p21 expression through interaction with the CSL/RBPJ-p53 complex, thereby facilitating cell cycle arrest (Chen et al., 2019). TGF-β signaling also targets OXPHOS (Table 1A). In murine and human natural killer cells, TGF-β signaling dampens cell metabolism and represses OXPHOS (Viel et al., 2016; Zaiatz-Bittencourt et al., 2018; Slattery et al., 2021), in a mTOR signaling-dependent (Viel et al., 2016) or -independent manner (Zaiatz-Bittencourt et al., 2018). In addition, TGF-β suppresses the activity of ATP synthase in effector memory CD4^+^ T cells and therefore reduces mitochondria respiratory capacity (Dimeloe et al., 2019). Since mitochondria are critical to many key immune functions (Mills et al., 2017), these inhibitory effects on OXPHOS in immune cells may underlie some negative effects of TGF-β in immunity. In mink lung epithelial Mv1Lu cells, TGF-β inhibits mitochondria complex IV activity and increases intracellular ROS accumulation, leading to senescence (Yoon et al., 2005). However, TGF-β has also been reported to enhance OXPHOS. For instance, in MCF-7 breast cancer cells, TGF-β increases the expression of OXPHOS-associated proteins, including NADH:ubiquinone oxidoreductase subunit B8 (NDUFB8), cytochrome c oxidase subunit I (COX I) and mitochondrial transcription factor A (TFAM) during EMT, a cellular process that is thought to promote metastasis (Liu et al., 2020). In addition, TGF-β signaling in precursors of exhausted effector T cells promotes OXPHOS by repressing mTOR, enabling the preservation of mitochondrial metabolism that supports long-term T cell responses during chronic infection (Gabriel et al., 2021).

Aerobic glycolysis, or the Warburg effect, is widely adopted in many cancer cells (Hanahan and Weinberg, 2011), which is characterized by the preference of glycolysis over oxidative phosphorylation as a major source of energy production even when oxygen is abundant. Aerobic glycolysis can be induced in normal mammary fibroblasts by overexpression of constitutively active TβRI, powering the metabolically reprogrammed fibroblasts to fuel growth of cancer cells via energy transfer (Guido et al., 2012; Martinez-Outschoorn et al., 2012). In prostate cancer cells, overexpression of Smad2/3 enhances aerobic glycolysis independently of TGF-β stimulation but requires PKCε-mediated phosphorylation of the Smad3 linker region, which assists binding of Smad3 to the promoter of glycolytic genes (Xu et al., 2018). However, most of the studies were carried out in cell lines, and whether endogenous activation of TGF-β signaling promotes aerobic glycolysis in tumor cells awaits further investigation.

Compared to glucose catabolism, TGF-β1 has been documented to increase gluconeogenesis via the c-Jun/G6PC3 (glucose-6-phosphatase catalytic subunit 3) axis in zebrafish embryos, which fosters the nascent hematopoietic stem cells (Zhang et al., 2018). It would be worth exploring whether this mechanism can be applied to mammals or humans. Furthermore, there are many other metabolic pathways other than glycolysis that require glucose, including the pentose phosphate pathway, the hexosamine pathway, glycogenesis, the serine biosynthesis pathway and its many branches (Hay, 2016). Whether TGF-β signaling interacts with these pathways is unclear.

## TGF-β Signaling and Lipid Metabolism

Lipids are a large group of water-insoluble molecules that, according to their diverse cellular functions, can be roughly divided into three categories represented by triglycerides that store energy; phosphoglycerides, sphingolipids and sterols that build the main structure of biological membrane; and many derivatives that actively engage in signal transduction and enzymatic reaction (Ridgway and McLeod, 2008).

Fatty acids can be released from triglycerides and provide the energetic needs through fatty acid oxidation (β-oxidation) in mitochondria. Blocking TGF-β signaling in mice via Smad3 ablation promotes brown adipogenesis within white adipose tissue and boosts mitochondria biogenesis in adipocytes, causing a significant elevation in fatty acid oxidation (Yadav et al., 2011). Conditional knockout of *Tgfbr2* in hepatocytes ameliorates CDAA (choline-deficient l-amino acid-defined) diet-induced steatohepatitis in mice, prevents CDAA-induced expression of genes related to lipogenesis, and enhances gene expression involved in β-oxidation (Yang et al., 2014). As inhibition of TGF-β signaling promotes fatty acid oxidation, it is plausible to postulate that TGF-β signaling activates the synthesis of fatty acids. Indeed, all three types of the TGF-β ligands, but not other members of the TGF-β superfamily, are shown to increase stearoyl-CoA desaturase expression in a Smad-dependent way in many human cell lines (Samuel et al., 2002). However, other studies demonstrate that the effect of TGF-β on fatty acid oxidation or synthesis is context-dependent (see Table 1B for details). Many studies report that TGF-β suppresses the expression of fatty acid synthase during the induction of EMT in cancer cells (Jiang et al., 2015; Yang et al., 2016; Liu et al., 2020). In Hep3B cells, TGF-β causes a significant reduction in carnitine-conjugated fatty acids, which coincides with upregulation of fatty acid transporter genes, implying increased carnitine-mediated entry of fatty acids into mitochondria that are destined for β-oxidation (Soukupova et al., 2021). In addition, TGF-β2 or TGF-β3, but not TGF-β1, is shown to reinforce fatty acid oxidation in myotubules and adipocytes (Takahashi et al., 2019). Ketone bodies are formed in the liver from acetyl-CoA produced by oxidation of fatty acids. As a major form of ketone body, β-hydroxybutyrate has been reported to increase TGF-β expression in HK-2 renal cells (Guh et al., 2003). However, the effect of TGF-β on the ketone bodies remains unknown.

**FIGURE 3:**
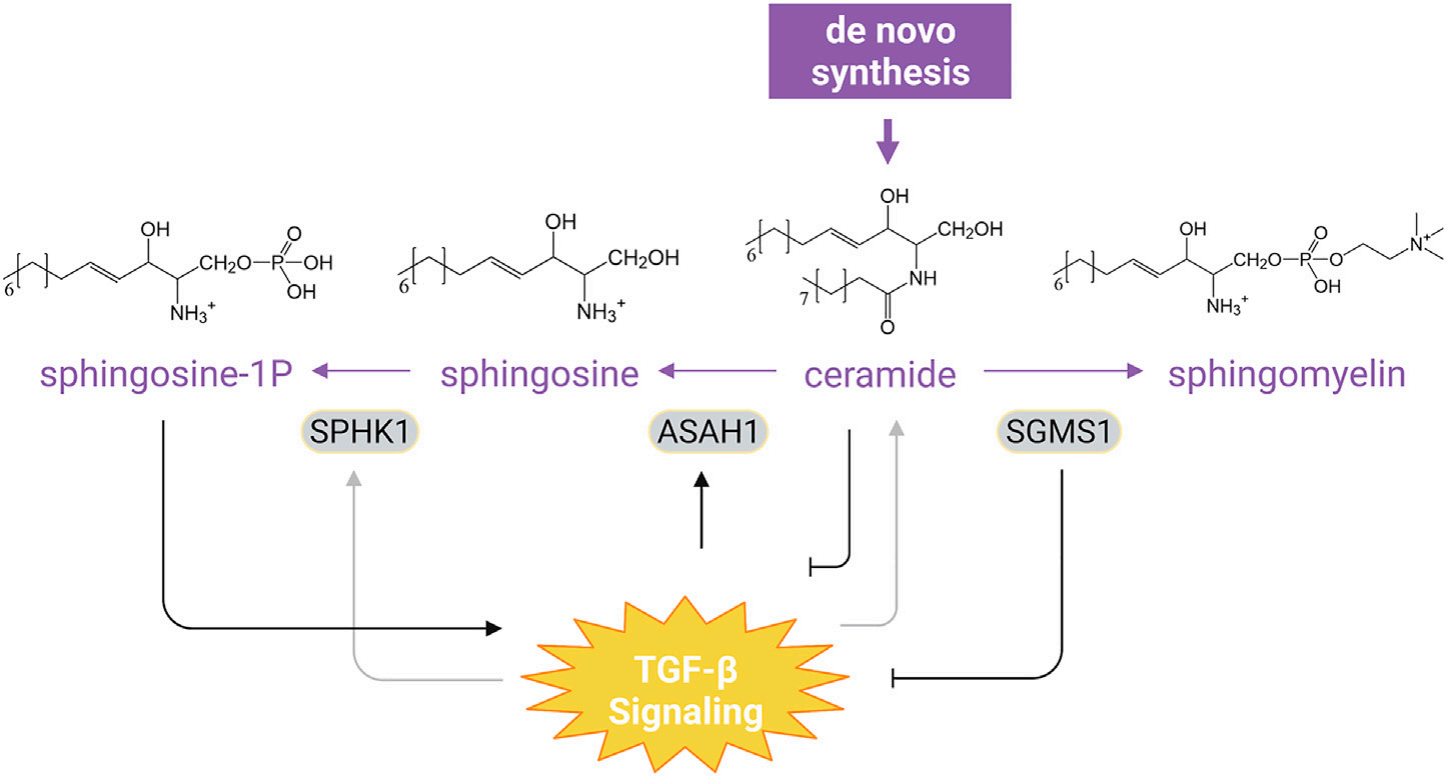
Interaction of TGF-β Signaling with Sphingolipid Metabolism. TGF-β differentially regulates SPHK1 expression in a context-dependent manner and can upregulate ASAH1 to promote aberrant accumulation of sphingosine. Ceramide, the only sphingolipid that can be *de novo* synthesized, has been shown to constrain TGF-β signaling. Overexpression of SGMS1, which catalyzes synthesis of sphingomyelin from ceramide, also inhibits TGF-β signal transduction. Moreover, sphingosine-1P can evoke TGF-β-like responses in cells (see text). For simplicity, synthesis of other sphingolipid and the downstream catabolism of sphingosine-1P have been omitted. Purple texts and arrows, the sphingolipid metabolic pathway; gray arrows, cell-type dependent effect. Abbreviations: SPHK1, sphingosine kinase 1; ASAH1, N-acylsphingosine amidohydrolase 1; SGMS1, sphingomyelin synthase 1. Figure is created with BioRender.com.

TGF-β signaling also regulates the metabolism of some structural lipids that define the membrane architecture (Table 1B). Sphingolipids are a large class of membrane lipids, among which ceramide is the only one that can be *de novo* synthesized and serves as the structural precursor of higher sphingolipid members (Figure 3). Ceramide can be hydrolyzed by N-acylsphingosine amidohydrolase 1 (ASAH1) into sphingosine, which can be phosphorylated by sphingosine kinase 1/2 (SPHK1/2) into sphingosine-1-phosphate (S1P) to regulate a variety of physiological and pathological processes (Maceyka et al., 2012). In NMuMG cells and human normal bladder HCV29 cells, TGF-β can rewire glycosphingolipid composition to promote EMT by reducing intracellular levels of gangliotetraosylceramide or GM2 (Guan et al., 2009). TGF-β enhances the activity and expression of SPHK1 in human fibroblasts that are important for the expression of TIMP-1 (Yamanaka et al., 2004). In contrast, TGF-β1 and TGF-β3 downregulates SPHK1 expression but upregulates ASAH1 expression in the human choriocarcinoma JEG-3 cell line, leading to aberrant sphingosine accumulation characteristic of dysfunctional placentae in intrauterine growth restriction (Chauvin et al., 2015). TGF-β can also diminish ceramide production to inhibit apoptosis in NIH3T3 cells during serum starvation (Chen et al., 2003), while increasing ceramide levels in human dermal fibroblasts and Mv1Lu cells (Sato et al., 2003). The increased ceramide is shown to act as a positive regulator of TGF-β signaling by facilitating TGF-β-induced COL1A2 expression in foreskin fibroblasts (Sato et al., 2003). Ceramide has also been reported to inhibit TβRI/II trafficking to primary cilia by stabilizing the TβRI-Smad7 interaction, thereby attenuating cell migration and metastasis (Gencer et al., 2017). Consistent with the observation that TGF-β induces SPHK1 expression, exogenous sphingosine 1-phosphate can elevate phosphorylated Smad2 levels and increase TIMP-1 expression in rat renal mesangial cells (Yamanaka et al., 2004). Moreover, overexpression of sphingomyelin synthase 1, a key enzyme that converts ceramides into sphingomyelins, downregulates TβRI expression and thus impairs TGF-β-induced EMT in breast cancer cell lines (Liu et al., 2019). Aside from sphingolipid metabolism, the metabolic pathway of cholesterol has been shown to regulate TGF-β signaling (Table 2B). Cholesterol is enriched in lipid rafts, a membrane microdomain which modulates TGF-β signaling. TGF-β receptors can be internalized via lipid raft-dependent endocytosis and transported to lysosome for degradation (Chen, 2009), while the location at lipid rafts of TGF-β receptors is required for TGF-β activation of MAP kinases (Zuo and Chen, 2009). Cholesterol depletion specifically inhibits TGF-β-induced activation of extracellular signal-regulated kinase (ERK) and p38 and therefore impairs EMT and cell migration (Zuo and Chen, 2009). In addition, loss of the rate limiting enzyme Nsdhl (NAD(P)-dependent steroid dehydrogenase-like) involved in cholesterol synthesis in mouse pancreatic ductal adenocarcinoma cells activates *Srebp1* (sterol regulatory element-binding protein 1), which enhances TGF-β1 expression and secretion and consequently facilitates EMT (Gabitova-Cornell et al., 2020). However, another study reported an opposite observation in human breast cancer cells: NSDHL expression augments TGF-β signaling by inhibiting TβRII degradation and therefore promotes cell migration (Chen et al., 2021). Hence, like in many other cases, this regulation is cell-specific.

The phosphorylated derivatives of phosphatidylinositol (PI) play a critical role in intracellular signal transduction. Phosphorylation of PI(4, 5)P_2_ to PI(3, 4, 5)P_3_ by PI-3-kinase triggers activation of Akt, inhibiting apoptosis and promoting cell survival (Manning and Toker, 2017). In hematopoietic cells, TGF-β signals are shown to counteract Akt signaling and promote apoptosis by upregulating the expression of the SH2-containing inositol phosphatase SHIP (Valderrama-Carvajal et al., 2002), which breaks down PI(3, 4, 5)P_3_ to PI(3, 4)P_2_. The interplay between TGF-β signaling and lipophilic hormones such as retinoic acid and vitamin D is well studied (Tables 1B, 2B). Retinoic acid has been shown to synergistically increase the expression and phosphorylation of Smad3 in the presence of TGF-β during differentiation of CD4^+^ T cells toward Treg (Xiao et al., 2008). The biologically active form of vitamin D, 1α,25-dihydroxyvitamin D_3_ (1,25(OH)_2_D_3_), has been reported to revert TGF-β-increased OXPHOS and reactive oxygen species (ROS) in human bronchial epithelial cells (Ricca et al., 2019). 1,25(OH)_2_D_3_ has also been shown to antagonize TGF-β-mediated fibrogenesis. In the presence of the ligands, the vitamin D receptor (VDR) occupies Smad3-binding sites at profibrotic genes and reduces TGF-β-mediated hepatic fibrosis (Ding et al., 2013). Similarly, VDR ablation abolishes the antagonistic effect of 1,25(OH)_2_D_3_ on TGF-β-promoted hepatic fibrosis (Beilfuss et al., 2015). In human leiomyoma cells, 1,25(OH)_2_D_3_ can reduce Smad2 expression or activation by TGF-β and thus expression of profibrotic genes (Halder et al., 2011). In hepatic stellate cells, vitamin D supplementation also showed similar effects (Beilfuss et al., 2015). Reversely, TGF-β can cause vitamin D catabolism through upregulation of the vitamin D-24A-hydroxylase CYP24A1, resulting in undermined host defense in airway epithelium (Schrumpf et al., 2020). Interestingly, Smad3 can form a complex with VDR in a ligand-dependent manner and enhances its transactivation activity (Yanagisawa et al., 1999).

Lipid droplets (LD) are a type of organelle instrumental in lipid and energy homeostasis and also involved in diverse cellular activities other than lipid metabolism (Olzmann and Carvalho, 2019; Walther and Farese, 2012). TGF-β has been demonstrated to induce its formation in many cell types (Table 1B). TGF-β2 induces fatty acids storage and LD formation in acidosis-adapted cancer cells, which meets cellular energetic needs for EMT and cell invasion (Corbet et al., 2020). It also increases LD content in dendritic cells under acidic circumstances (Trempolec et al., 2020). In addition, treatment of murine macrophages with TGF-β causes LD accumulation, accompanied by a shift of macrophages from M1 phenotype to the pathological M2 phenotype (Bose et al., 2019). However, the mechanisms underlying TGF-β-induced LD formation are currently unclear.

## TGF-β Signaling and Amino Acid Metabolism

It has come to appreciate that amino acids, besides their fundamental role as substrates for protein synthesis, also perform multifarious cellular functions including energy homeostasis, cell growth and immune response (Wu, 2009). Taking advantage of metabolomics, Nakasuka and others have nicely demonstrated that TGF-β can change intracellular amino acid levels in non-small cell lung cancer cells (Nakasuka et al., 2021). Depletion of a particular amino acid (e.g., Phe, Thr, Leu, Ile, or Tyr), whose intracellular concentrations are significantly decreased by TGF-β, in culture media, induces EMT-like elongated morphology. They further showed that TGF-β induces the expression of prolyl 4-hydroxylase subunit alpha 3, an enzyme catalyzing proline to 4-hydroxylproline, whose knockdown abrogates TGF-β-induced amino acid changes and EMT (Nakasuka et al., 2021). It would be intriguing to know how altered expression of one gene involved in proline metabolism can cause global changes of amino acid levels.

In addition to its comprehensive effects on amino acid metabolism, TGF-β signaling also specifically mediates certain amino acid metabolic pathways (Table 1C). For instance, TGF-β modulates glutamine metabolism, which takes a key part in tumor development (Zhang et al., 2017a). In hepatocellular carcinoma cells, TGF-β augments glutamine metabolism by inducing the expression of glutamine transporter and glutaminase 1 (GLS1) and reduces oxidative metabolism, concomitant with enhanced EMT and cell migration (Soukupova et al., 2017). Interestingly, the way TGF-β induces *GLS1* expression seems to cell type-specific. In MCF-7 cells, TGF-β-induced *GLS1* expression is mediated by the transcription factor Dlx-2, leading to enhanced glutamine metabolism that contributes to EMT (Lee et al., 2016). In myofibroblasts, however, TGF-β upregulates *GLS1* expression via Smad3 and p38 and promotes myofibroblast differentiation (Bernard et al., 2018). Furthermore, TGF-β elevates GLS1 levels in AKR-2B mouse fibroblasts by repressing the transcription factor SIRT7 and FOXO4, and the process requires Smad2/3 as well as mTOR (Choudhury et al., 2020).

Tryptophan metabolism, especially in immune cells, exemplifies another aspect of amino acid metabolism modulated by TGF-β signaling (Table 1C). At the core of tryptophan metabolism lies the kynurenine pathway, in which kynurenine is generated from tryptophan, serving as the common precursor for the synthesis of various downstream metabolites including NAD^+^ (Kolodziej et al., 2011). Two serial enzymatic reactions convert tryptophan to kynurenine, and the first and rate-limiting step is catalyzed by three different enzymes: IDO-1 (indoleamine 2,3-dioxygenase-1), IDO-2 (indoleamine 2,3-dioxygenase-2) or TDO (tryptophan 2,3-dioxygenase). TGF-β prominently abolishes IFN-γ-induced IDO expression in human fibroblasts (Yuan et al., 1998). In contrast, IDO expression is upregulated by TGF-β in dendritic cells, which relies on the TGF-β-induced expression of arginase 1 and increased abundance of spermidine (Mondanelli et al., 2017). Importantly, TGF-β also confers IDO immunoregulatory function independently of its metabolic activity. By inducing phosphorylation of IDO at the putative immunoreceptor tyrosine-based inhibitory motifs (ITIMs) via the kinase Fyn, TGF-β promotes the complex formation of IDO with two tyrosine phosphatase SHP-1 and SHP-2, thereby activating a circuit of downstream signaling events required to maintain self-tolerance (Pallotta et al., 2011).

TGF-β also regulates other amino acid metabolic pathways (Table 1C). In human lung fibroblasts, TGF-β activates expression of ATF4, a master transcription factor of amino acid metabolism (Ameri and Harris, 2008; Kilberg et al., 2009), and leads to upregulation of *PHGDH*, *PSAT1*, *PSPH* and *SHMT2*, which are key players involved in glycine-serine synthesis (Nigdelioglu et al., 2016; Selvarajah et al., 2019). TGF-β inhibits leucine transporter SLC3A2 expression and therefore impairs leucine uptake, contributing to TGF-β-induced cell cycle arrest of mammary epithelial cells (Loayza-Puch et al., 2017). In NIH3T3 fibroblasts, the TGF-β/Smad signaling stimulates proline synthesis from glutamate by elevating protein levels of pyrroline-5-carboxylate synthase and pyrroline-5-carboxylate reductase 1/2 in the synthetic pathway to buffer mitochondrial redox stress (Schworer et al., 2020).

## TGF-β Signaling and Other Aspects of Cell Metabolism

Homeostasis of redox metabolism is crucial to an extensive range of cellular and physiological conditions. The redox imbalance, often arises from aberrant accumulation of ROS and is marked by oxidative stress, can promote progression of multiple diseases (Sies et al., 2017). The crosstalk between redox metabolism and TGF-β signaling during cancer and fibrosis is comprehensively reviewed elsewhere (Richter et al., 2015; Ramundo et al., 2021). Noteworthy, the enzyme NADPH oxidase 4 (NOX4) appears to play a main role in mediating TGF-β-induced ROS generation under many circumstances (Cucoranu et al., 2005; Sturrock et al., 2006; Carmona-Cuenca et al., 2008; Michaeloudes et al., 2011; Boudreau et al., 2012). TGF-β can also inhibit the key antioxidant systems by downregulating glutathione (GSH) metabolism (Liu and Gaston Pravia, 2010). However, increased GSH metabolism and alleviated ROS levels are also observed in TGF-β-mediated drug resistance of squamous cell carcinoma cells (Oshimori et al., 2015).

**FIGURE 4:**
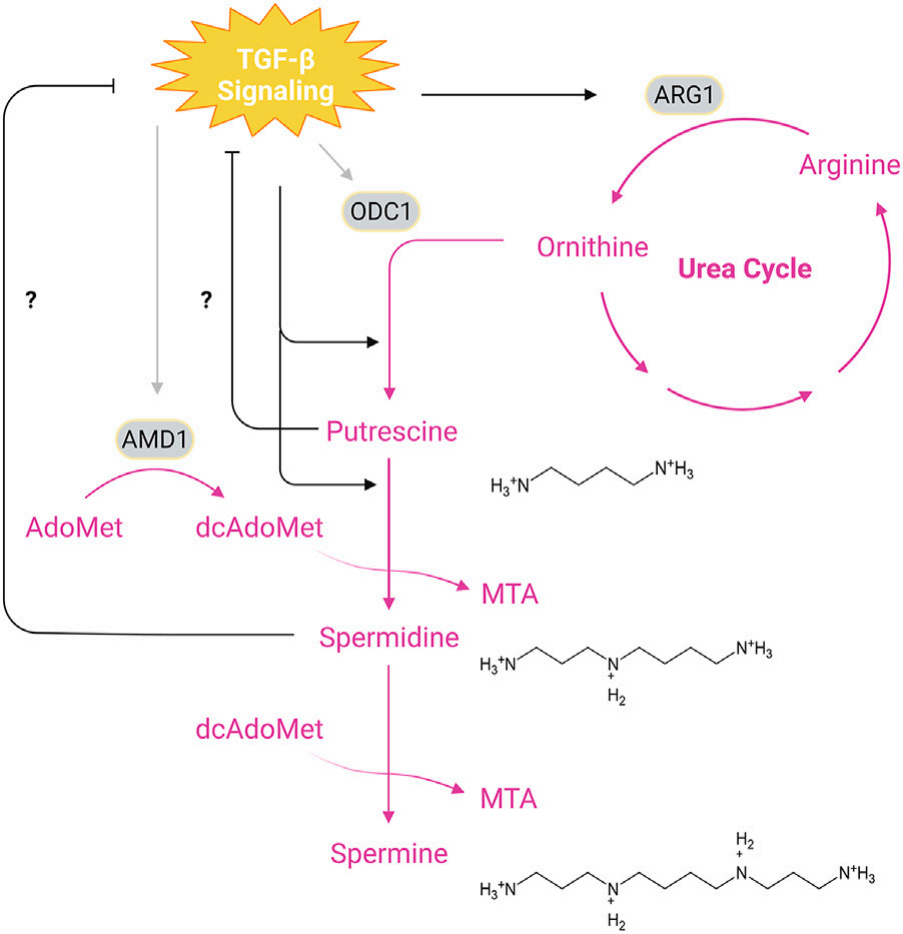
Interplay Between TGF-β Signaling and Polyamine Synthesis. The main biosynthetic pathway of polyamines begins with ODC1-catalyzed formation of putrescine from ornithine, a product of the urea cycle, which is generated from arginine through hydrolysis by ARG1. Synthesis of spermidine and spermine requires transfer of aminopropyl groups from dcAdoMet, a decarboxylated product of AdoMet (S-adenosylmethionine) catalyzed by AMD1. Putrescine and spermidine may inhibit TGF-β signaling since depletion of cellular putrescine and spermidine has been shown to enhance TβRI/II levels and Smad nuclear translocation, while TGF-β signaling promotes synthesis of putrescine and spermidine through upregulation of ARG1. In addition, TGF-β signals can stimulate or dampen the activity of ODC1 and AMD1 depending on the cell types. Pink texts and arrows, the urea cycle and polyamine synthesis. Abbreviations: MTA, methylthioadenosine; ARG1, arginase 1; ODC1, ornithine decarboxylase 1; AMD1, adenosylmethionine decarboxylase 1. Figure is created with BioRender.com.

The polyamine metabolic pathway attracts great interests in the past decade due to their roles in cell biology beyond early described importance for cell proliferation (Miller-Fleming et al., 2015). Depending on the cell types, TGF-β signaling can differentially regulate the activity of the two rate-limiting enzymes ornithine decarboxylase (ODC1) and adenosylmethionine decarboxylase 1 (AMD1) in polyamine synthesis (Figure 4; Table 1D). TGF-β suppresses the enzymatic activity of ODC1 and AMD1 in leukemic cells (Motyl et al., 1993), while stimulating their activities in myofibroblasts (Blachowski et al., 1994). TGF-β can also indirectly regulates the polyamine synthesis in immune cells and vascular smooth muscle cells. By inducing the expression of arginase 1, an enzyme that converts arginine to ornithine that serves as the common precursor for polyamine synthesis, TGF-β is able to increase the arginine-dependent production of specific polyamines (Mondanelli et al., 2017; Boutard et al., 1995; Durante et al., 2001). However, a recent study reported that the TGF-β blunt the increased influx of arginine to putrescine and spermidine during polarization of CD4^+^ naïve T cells (Puleston et al., 2021). Polyamine metabolism can also modulate the TGF-β signal transduction (Table 2C). In mouse intestinal epithelial cells, depletion of intracellular polyamines by an ODC1 inhibitor DFMO leads to increased expression of TGF-β, TβRI, Smad3 and Smad4 as well as nuclear accumulation of these Smads, sensitizing cells to TGF-β-induced cytostasis (Patel et al., 1998; Rao et al., 2000; Liu et al., 2003).

In addition, TGF-β is able to upregulate the expression of *PNPO* (pyridoxamine 5′-phosphate oxidase), which encodes the rate-limiting enzyme in vitamin B6 metabolism, to produce active forms of vitamin B6 that may promote ovarian cancer progression (Zhang et al., 2017b). Adenosine secreted from myeloid cells is shown to modulate TGF-β signaling in proximal fibroblasts by reducing phosphorylation of Smad2/3 and to affect ECM deposition and therefore influence the tumor microenvironment of mammary carcinoma (Vasiukov et al., 2020). Furthermore, downregulation of the purine catabolism enzyme xanthine dehydrogenase increases TGF-β2/3 and phosphorylated Smad2/3 levels and contributes to EMT and cell migration in hepatocellular carcinoma cell lines (Chen et al., 2017). How these metabolic alterations convey their regulatory instructions to TGF-β signaling awaits further investigation.

## Concluding Remarks

As summarized above, TGF-β signaling can exert its cellular and physiological effects through reprograming of cell metabolism. It controls the activity of many metabolic pathways as wells as the production of functional metabolites by regulating the expression of key metabolic proteins or enzymatic activities (Motyl et al., 1993; Blachowski et al., 1994; Yoon et al., 2005; Dimeloe et al., 2019; Hua et al., 2020; Li et al., 2020; Smith and Hewitson, 2020). In addition, TGF-β signaling is able to reprogram cell metabolism by conferring enzymes non-metabolic functions through post-translational modification (Pallotta et al., 2011; Chen et al., 2019). Of note, the metabolic outputs of TGF-β signaling in cells are context-dependent and highly specific to the cell type, which probably result from the different epigenetic landscapes of distinct cell types, or the different Smad-interacting transcriptional cofactors (Feng and Derynck, 2005; Massague, 2012; David and Massague, 2018). Importantly, rather than being passively regulated by TGF-β signaling, cell metabolism can also modulate TGF-β signaling. Intracellular metabolites and metabolic proteins affect the production or bioactivity of the TGF-β ligands, influence the expression and membrane levels of TGF-β receptors (Rao et al., 2000; Wu and Derynck, 2009; Gencer et al., 2017; Liu et al., 2019; Gabitova-Cornell et al., 2020; Chen et al., 2021), regulate phosphorylation or the abundance of Smad proteins (Inoki et al., 1999; Xiao et al., 2008; Halder et al., 2011; Beilfuss et al., 2015; Chen et al., 2017; Vasiukov et al., 2020), and impact translocation of Smad complex or their binding to TGF-β-target genes (Liu et al., 2003; Ding et al., 2013).

Despite reasonable knowledge have been acquired to date, many questions about the interconnection between TGF-β signaling and cell metabolism still remain. First, we lack a characterization of TGF-β-responsive metabolic gene signature across different cell types, and we do not know how many metabolites or metabolic enzymes can also function as signaling effectors in response to TGF-β. A combination of transcriptomics, untargeted metabolomics and phosphoproteomics will considerably aid in handling this problem. Second, the underlying mechanisms by which metabolites regulates TGF-β signaling remain poorly understood. Since control of gene expression appears to be a mainstay of metabolite-mediated regulation of TGF-β signaling, it would be worthy to investigate if epigenetic regulation by metabolites could account for their modulatory effects (Li et al., 2018). Last, the majority of experiments were conducted *in vitro* using cell lines and whether these findings could be reproduced at a physiological level are currently unknown. Hence, it is of great importance to develop mouse models to examine if the interactions between TGF-β and cell metabolism are indeed physiologically and pathologically relevant. These emerging problems at the interface between TGF-β signaling and cellular metabolism might offer new avenues for future research and bring therapeutic benefits to treat diseases.
